# Effects of Additives on Silage Fermentation Characteristic and In Vitro Digestibility of Perennial Oat at Different Maturity Stages on the Qinghai Tibetan

**DOI:** 10.3390/microorganisms9112403

**Published:** 2021-11-22

**Authors:** Ping Li, Xiaolong Tang, Chaosheng Liao, Maoya Li, Liangyin Chen, Guangrou Lu, Xiaokang Huang, Chao Chen, Wenlong Gou

**Affiliations:** 1 College of Animal Science, Guizhou University, Guiyang 550025, China; lip@gzu.edu.cn (P.L.); T18085687191@163.com (X.T.); L17785468695@163.com (C.L.); lychen2011430@sina.com (L.C.); L18185450732@163.com (G.L.); H15329727872@163.com (X.H.); 2 Sichuan Academy of Grassland Sciences, Chengdu 611731, China; Y18385865040@163.com

**Keywords:** perennial oat, lactic acid bacteria, silage fermentation, in vitro digestibility, aerobic stability

## Abstract

To effectively use local grass resources to cover the winter feed shortage on the Qinghai-Tibetan Plateau, the silage fermentation and in vitro digestibility of perennial oat (*Helictotrichon* *virescens* Henr.) were investigated. Perennial oat was harvested at the heading/flowering stage, wilted under sunny conditions, chopped, vacuumed in small bag silos, and stored at ambient temperatures (5–15 °C) for 60 days. The silages were treated without (CK) or with local lactic acid bacteria (LAB) inoculant (IN1), commercial LAB inoculant (IN2), and sodium benzoate (BL). Control silages of perennial oat at early heading stage showed higher (*p* < 0.05) lactate and acetate contents and lower (*p* < 0.05) final pH, butyrate, and ammonia-N contents than those at the flowering stage. High levels of dry matter recovery (DMR) and crude protein (CP) were observed in IN1- and BL-treated silages, with high in vitro gas production and dry matter digestibility. Compared to CK, additives increased (*p* < 0.05) aerobic stability by inhibiting yeasts, aerobic bacteria, and coliform bacteria during ensiling. In particular, the local LAB inoculant increased (*p* < 0.05) concentrations of lactate, acetate and propionate, and decreased concentrations of butyrate and ammonia-N in silages. This study confirmed that local LAB inoculant could improve the silage quality of perennial oat, and this could be a potential winter feed for animals such as yaks on the Qinghai Tibetan Plateau.

## 1. Introduction

Grazing yaks on the Qinghai Tibetan Plateau suffer from starvation due to feed storage in the winter and early spring. Otherwise, yaks (especially during the fatting period) are displaced in warmer regions with abundant feed resources. However, the long-distance transport from a cold region to a warmer region may be costly for famers and unfavorable for the yak’s health. Meanwhile, a short growing season with unstable climates may increase the unavailability of traditional forages such as oat. Silage has become an increasing important source of yak feed on the Qinghai Tibetan Plateau. Suitable plants for silage production mainly include perennial grass, annual grass, and crops in this region [1,2,3,4]. Some cultivated grasses have attracted attention for silage making due to their relatively high yields and nutritive value under unfavorable climatic conditions. Perennial oat (*Helictotrichon* *virescens* Henr.) is mainly distributed Asia, Europe, and North America [5]. Based on the laboratory and field measurements conducted in our study, the dry matter yields of perennial oat is 6.9~13.6 t/hm^2^ on the Qinghai Tibetan Plateau, with a CP of 8.8% DM, crude fat of 1.9% DM, crude fiber of 3.13% DM, and ash of 6.4% DM at the flowering stage. Perennial oat could be grazed by livestock or cultivated for silage production.

Ensiling is a traditional and convenient method that can be used to preserve the nutrients of forages. During ensiling, lactic acid bacteria (LAB) convert water soluble carbohydrates (WSC) to organic acids (mainly lactic acid) for quick pH decline (below 4.6) to inhibit the cell activities of plants and microbes. In a low temperature environment, the silage fermentation is usually impaired or unfinished due to the low numbers of desirable microorganisms on the plants [6]. This situation is worsened by the unpredictability of the weather and the wilting conditions during ensiling on the Qinghai Tibetan Plateau [2,7]. Microbial additives are widely used to promote adequate fermentation and to preserve the nutritive components of silages and may be an effective tool for silage production in cold regions [8]. However, some commercial LAB inoculants from temperate regions have exhibited a poor effect on silage fermentation on the Qinghai Tibetan Plateau [3]. The beneficial effects of local LAB on the fermentation of silages on the Qinghai Tibetan Plateau have been reported by Zhang et al. (2017) [8], Wang et al. (2017) [9], and Chen et al. (2020) [2,3]. Bernardes et al. (2018) reviewed that although the use of bacterial inoculants as silage additives has increased in Northern Europe, acid-based additives are still a good option, especially in difficult weather conditions, to ensure good fermentation quality as well as a high intake potential and nutritive value of the silages [6]. To enhance aerobic stability, new antifungal additives such as sodium benzoate have been designed and evaluated in different silages [10,11,12]. In addition, chemical additives such as sodium benzoate may be effective in enhancing the digestibility and anaerobic stability of silage [10]. However, little information is available on the additive-treated perennial oat silage.

Therefore, the objective of this study was to evaluate the effects of chemical and microbial additives on silage fermentation and on the in vitro ruminal digestibility of perennial oat grass on the Qinghai Tibetan Plateau. We supposed that (1) perennial oat at different growing stages could be ensiled well; (2) the silage quality of perennial oat could be enhanced by applications of chemical and microbial additives at ensiling; and (3) local LAB inoculant exhibited good performance in improving silage fermentation, aerobic stability, and in vitro gas production.

## 2. Materials and Methods

### 2.1. Silage Making

Six plots (15 m^2^, each) were seeded with perennial oat with the density needed for the experiment base at the Sichuan Academy of Grassland Science, which is located on the southeast edge of Qinghai-Tibetan Plateau (N 31°51′–33°33′, E 101°51′–103°22′, altitude 3500 m; Hongyuan, Zhejiang, P.R. China). In the second year, the perennial oat from each plot was harvested manually above ground when it had reached a height of 5 cm at the early heading stage (three plots) and flowering stage (three plots); it was wilted for 2 h under sunny conditions and chopped at length of 1–2 cm. The chopped perennial oat from each plot was randomly divided into four equal parts for the following treatments: (1) no additives (CK); (2) local LAB inoculant (IN1; *Lactobacillus plantarum* BP18, *Pediococcus* *pentosaceus* HS1 and *L. buchneri* LP22; isolated from natural fermented silages on the Qinghai Tibetan Plateaus; applied at 10^6^ cfu/g FM, recommend by Chen et al. (2020) [2]; (3) commercial LAB inoculant (IN2; *L. plantarum*, *L. buchneri*, each 10^6^ cfu/g FM, and provided Gaofuji Biotechnology Co., Ltd., Chengdu, China); and (4) chemical additive (BL; sodium benzoate, applied at an optimal rate of 2.4 g/FM, recommended by the EFSA Panel on Additives and Products or Substances used in Animal Feed [13]). All of the chopped grasses were well-mixed additives that were manually placed into plastic bags (500 g, each bag), degassed by a vacuum sealer, and stored in dark room at ambient temperatures 5–15 °C for 60 days.

### 2.2. Chemical Analysis

A fresh sample (20 g) from each bag silo was mixed with 180 mL distilled water for 3 min in a Stomacher blender. The pH of the filtrate was determined using a pH meter (PHSJ-4 F; Shanghai INESA Scientific Instrument Co., Ltd., Shanghai, China). Filtrate of about 5 mL was subjected to centrifugation (4500× *g*, 15 min, 4 °C), and the supernatant was analyzed for lactate, acetate, propionate, and butyrate using high-performance liquid chromatography [14]. Ammonia-N was determined according to the method of Broderick and Kang (1980) [15].

Freeze-dried samples were ground with a mill (1 mm screen). Crude protein (CP) was analyzed by AOAC (2000) and was calculated using the formulation Kjeldahl N × 6.25 [16]. The neutral detergent fiber (NDF) and acid detergent fiber (ADF) were determined by the methods of Van Soest et al. (1991) using an Ankom 2000 fiber analyzer (Ankom Technology Co., Ltd., Macedon, NY, USA) [17]. WSC was determined according to the method of McDonald et al. (1991) [18]. Dry matter recovery and aerobic stability from the silage was determined according to the methods of Kung et al. (2018) [10].

### 2.3. Microbial Analysis

Samples of the silage materials that were 10 g in size were shaken well with 90 mL of distilled water, and serial dilutions from 10^−^^3^ to 10^−5^ were prepared in distilled water. LAB were counted on MRS medium agar after incubation at 30 °C for 48 h in an CO2 incubator (WCI-180 N, WIGGENS, Straubenhardt, Germany). The aerobic bacteria were counted on nutrient agar (Sangon Biotech Ltd., Shanghai, China), the yeasts and molds were counted on potato dextrose agar (Qingdao Hope Bio-Technology Co., Ltd., Qingdao, China) with a sterilized 10% tartaric acid solution, and the coliform bacteria were counted on Blue Light Broth agar (Nissui-Seiyaku Ltd., Tokyo, Japan). These agar plates were incubated at 28 °C for 48 h. The colonies were counted as the numbers of viable microorganisms in colony-forming units (CFU)/g of fresh matter (FM).

### 2.4. In Vitro Digestibility

All experimental protocols were approved by the Review Committee for the Use of Human or Animal Subjects of Sichuan Academy of Grassland Sciences. The in vitro DM digestibility (IVDMD) of each sample from the silages was determined according to the method of Tilley and Terry (1963) [19]. In brief, the rumen fluid, collected from a healthy Yak fed with a diet as described by Chen et al. (2020) [3], was strained through double-layer gauze and mixed with a buffer solution as described by Menke and Steingass (1988) [20]. Each fresh sample (equal to about 0.2 g dry sample) was weighed into polypropylene synthetic tissue filter bags (5 × 5 cm in size with a pore size of 50 μm, provided from ANKOM Co., Ltd., Macedon, NY, USA) and was then put into serum bottles (250 mL) separately. The bottles were pre-warmed at 39 °C before the injection of the rumen fluid–buffer mixture (45 mL). The samples were placed on a rotary shaker in an incubator and were incubated in a 39 °C water bath. Gas production was determined using the ANKOM RFS Gas Production Measurement System (Ankom Technology Co., Ltd., Macedon, NY, USA). After the in vitro ruminal digestion process, the bags were gently rinsed with sterile water and dried at 75 °C for 48 h to determine IVDMD. Cumulative gas production data collected every 2 h were fitted to the model of Φrskov and McDonald (1979) [21]: Y = A_1_ + A_2_(1 − e^−Ct^), where Y is the gas production at time t; A_1_ is the gas production from the immediately soluble fraction (mL); A_2_ is the gas production from the insoluble fraction (mL); C is the gas production rate constant (mL/h); A_1_ + A_2_ is the potential gas production (mL); and t is the incubation time (h).

### 2.5. Statistical Analysis

Factorial analysis of variance was applied to evaluate the effects of the maturity stage (M), additives (A), and their interaction (M × A) on the silage parameters in the General Line Model of SPSS (SPSS 19.0 program SPSS Inc.). Differences were only considered significant when the probability level was lower than 0.05 (*p* < 0.05).

## 3. Results and Discussion

### 3.1. Chemical and Microbial Composition of Perennial Oat

It is well established that both LAB and WSC play an important role in initiating fermentation for the preservation of silage nutrients. Available substrates such as WSC may vary with the stage of development at harvest. A delayed harvest of the forage in fall can result in a significant increase in non-fiber carbohydrates (NFC) as ambient temperatures decrease in the most northern agricultural area of eastern Canada and eastern Canadian regions [6]. Table 1 shows that the WSC concentrations of 4.89–6.12% DM were sufficient for silage fermentation. Silage is well preserved when the epiphytic LAB reaches at least 10^5^ cfu/g FM on the plant [22]. Relative to the heading stage, perennial oat showed a low fiber (NDF and ADF) content at the flowering stage. This may be due to the high ratios of leaf to stem (data was not shown in article). The LAB count on the plant was below 10^5^ cfu/g FM at the sprouting sage of perennial oat and lowered at the flowering stage. Aerobic bacteria, coliform bacteria, and yeasts distributed heavily at both maturity stages, with counts of >10^5^ cfu/g FM. Similar results were from Chen et al. (2020) who reported that gramineous grass on the Qinghai Tibetan Plateau exhibited low LAB counts and high undesirable microorganisms due to the high UV radiation and low ambient temperatures [2,3]. These suggest that the silage fermentation may be enhanced by LAB associated inoculants.

### 3.2. Chemical and Microbial Compositions of Perennial Oat Silage

The chemical and microbial compositions of perennial oat silage are shown in Table 2 and Table 3. Acid-based additives such as formic acid are recommended during silage production in cold regions [6]. However, most commercial acid-based silage additives are corrosive to silos and are unfavorable for human and animals. Sodium benzoate, as a new antimicrobial agent, was positive for the preservation of CP in perennial oat silages. Kung et al. (2018) obtained a similar result [10]. LAB inoculants are widely used during silage production. In the current study, both of the LAB inoculants showed good performance for the preservation of CP in silage. A study from Reich and Kung (2010) has also shown that the CP were preserved well in LAB-inoculated silage due to a fast pH decline from the effective conversion of WSC to desirable fermentation acids. The fiber (NDF and ADF) contents of local LAB inoculant-treated silages were a little different from those in the control silage [23]. In contrast, a study from Chen et al. (2020) illustrated that the application of the same inoculant increased the NDF and ADF contents of the oat silage [2]. The DMR of biomass during ensiling is a worldwide challenge because it is highly relevant to the feedstock production costs and CO_2_ greenhouse gas emissions that are associated with biofuel production [24]. Low DMR occurred mainly due to the metabolism of yeasts during ensiling, which utilize soluble carbohydrates and produce ethanol [25]. A fast fermentation initiated by LAB could have quickly stirred pH decline for the reduction in the yeast growth rates. The local LAB inoculant increased the DMR of silage. However, Li et al. (2019) reported that the inoculation of hetero-fermentative LAB decreased the DMR of silage due to the conversion of available substrates into liquid and gases [26]. Notably, commercial LAB inoculant did not increase the DMR of silage compared to the control. These results confirmed that local LAB inoculant showed potential for producing high quality perennial oat silage.

### 3.3. Aerobic Stability and Microbial Population of Silage

The well-preserved silage could be attributed to the microorganism activity during ensiling. Most LAB species grow the most rapidly at temperatures between 27°C and 38°C during ensiling [2]. As the storage temperature decreases, the LAB population sustains high proportions, whereas the yeast population becomes predominant in the silage mass [27]. Thus, the yeasts were present in silages. Coliform bacteria and aerobic bacteria were reduced in additive-treated silages. This may be because of the quick pH reduction by LAB or the antimicrobial activity of sodium benzoate that was present in the silages. No difference in the LAB count was observed between the local and commercial inoculant-treated silages, but both had considerably lower LAB counts than control. Decreased trends were observed in the number of aerobic bacteria, coliform bacteria, and yeasts. The effect of the LAB inoculations on the microbial population is inconsistent. Most studies showed that LAB inoculation increased the LAB numbers of silage [28], but Ni et al. (2017) observed no significant difference between LAB inoculated and control silages [29]. In cold regions, most LAB inoculants showed negative effects on the counts of undesirable microorganisms such as yeasts [2,3]. The effect of sodium benzoate on the yeast counts of corn silage is dose-dependent. In the present study, the yeast count of the BL-treated silage showed the lowest levels of 2.40 log_10_ cfu/g FM at the heading stage and 2.33 log_10_ cfu/g FM at the flowering stage.

It is well-known that yeasts can cause aerobic deterioration during the feed-out stage and can reduce nutritional the value of silage. Kung et al. (2018) concluded that the high numbers of yeasts in silages are usually associated with high concentrations of ethanol, and their numbers are often inversely related to the aerobic stability of the silages [30]. Good silages should contain less than the number (10^6^ cfu) of yeasts per gram of wet silage [31]. In fact, the control silage deteriorated after aerobic exposure of 30.66 h from the sprouting stage and 35.64 h from the flowering stage. Compared with the control, both the LAB inoculants decreased the numbers of yeasts in the silages. Furthermore, the trend of aerobic stability was always consistent with the yeasts. The high presence of aerobic bacteria may advance silage deterioration. We attributed this situation to the inclusion of *L. buchneri* in the inoculants. The species *L. buchneri,* as a hetero-fermentative LAB, exerts a good role in enhancing the aerobic stability of silage due to the high production of acetate during ensiling [27]. The difference in the aerobic stability of silages was observed between local and commercial LAB inoculants. A study from Chen et al. (2020) showed that the exogenous LAB-inoculated silages were characterized by the main distribution of the yeasts on the Qinghai Tibetan Plateau [3]. The BL-treated silage also exhibited good aerobic stability. Numerous studies have proved that sodium benzoate used as silage additive decreased yeast counts and delayed aerobic deterioration [10].

### 3.4. pH and Fermentation Products of Perennial Oat Silage

The nutritive value and aerobic stability of silage are also correlated with fermentation parameters. In total, the silage of perennial oat at the sprouting stage exhibited higher concentrations of lactate and acetate, resulting in lower levels of final pH, butyrate, and ammonia compared to that at the flowering stage (Table 4). This was not in accordance with the situation that concentrations of lactic acid and volatile acids are usually inversely related to DM content [10]. The reason behind this unknown. The development of the inoculants that combine facultative and obligate heterofermentative LAB (eg. *L. plantarum* + *L. buchneri*) has the goal of achieving the benefits of both types of inoculants in one product [28], where facultative heterofermentative LAB control the early active fermentation period for suppressing undesirable microorganisms (eg. enterobacteria and clostridia) and thus reducing proteolysis and fermentation DM losses, whereas the obligate heterofermentative LAB could slowly convert lactic acid to acetic acid after the active silage fermentation period, raising pH and improving the aerobic stability of the silage. In current study, however, both LAB inoculants with *L. buchneri* decreased the silage pH of perennial oat at the flowering stage. This may be due to the higher concentration of lactate in LAB inoculant-treated silages compared to the control. At the sprouting stage of perennial oat, the local LAB inoculant showed similar trends in decreasing the pH and increasing the lactate of silage, but not differences existed in the pH and lactate concentration between the control and IN2-treated silages. We suspect that the exogenous species of *L. buchneri* effectively converted the lactate into acetate and propionate during the late stages of silage fermentation under a relatively high ambient temperature. Moreover, the IN1-treated silages showed lower levels of butyrate and ammonia-N due to the inhibition of clostridia under a pH of <4.6. It is well known that antifungal additives such as sodium benzoate can effective against the growth of clostridia and the productions of butyrate and ammonia-N during silage fermentation [28]. Importantly, there was no significant difference in butyrate or ammonia-N between the IN1-and BL-treated silages. These suggest that the local LAB inoculant could be used as an alternative to reduce the clostridial fermentation of perennial oat silage under low temperature conditions.

### 3.5. In Vitro Gas Production and DM Digestibility of Perennial Oat silage

An assessment of in vitro gas production (GP) is largely used to evaluate the nutritive value of ruminant feeds by incubating substrate in buffered rumen fluid [32]. The in vitro GP characteristics determined by the fermentation of the perennial oat silages are shown in Table 5. There was an interactive effect from the maturity stage and an additive on the gas productions of the immediately soluble fraction (A1), the insoluble fraction (A2), and the potential gas production (A1 + A2). Local and commercial LAB inoculants increased the parameters of A1, A2, and A1 + A2 during the in vitro ruminal fermentation from the silages of perennial oat at the heading stage. However, this positive effect only occurred in the IN1-treated silages of perennial oat at the flowering stage. We speculated that the relatively high ambient temperature at the heading stage of the forage helped the LAB inoculants preserve the available substrates (e.g., CP) for quick gas production. Furthermore, the inclusion of local LAB inoculant showed a high ability to enhance silage fermentation under low temperature conditions, and the resulting silages could provide more available substrates for ruminal fermentation. The increased levels of in vitro gas production parameters (A1, A2, A1 + A2and GP) could be attributed to the enhanced production of lactic acid and/or acetic acid and decreased levels of pH and ammonia-N in silage [3]. No significant difference in the gas production from the soluble substrates (A_1_) was observed between control and BL-treated silages, whereas higher in vitro gas production parameters (A_2_, A_1_ + A_2_ and GP) occurred to the BL-treated silages compared to the control. We speculated that the sodium benzoate-containing silage decreased the activity of the rumen microbes, and this short-term effect disappeared as the level that the microbes were able to adapt to increased during rumen fermentation. Differences in IVDMD depend on the nutritive components of the silages. In the present study, the IN1-and BL-treated silages had the highest IVDMD, which could be attributed to the increased DMR of the silages where more available substrates such as CP could be utilized during rumen fermentation.

## 4. Conclusions

Perennial oats could be used as a material for silage production on the Qinghai Tibetan Plateau. Both the local LAB inoculant and sodium benzoate showed better performance in the preservation of silage nutrients. In particular, the local LAB inoculant enhanced the silage fermentation of perennial oat at the heading and flowering stages, with high lactate and acetate concentrations and low pH, ammonia-N, and butyrate levels. Moreover, local LAB furthered the improvement in aerobic stability, DMR, and nutritive value with higher IVDMD and in vitro gas production. Thus, the local LAB inoculant was recommended for producing high quality silage of perennial oat under low ambient temperature.

## Figures and Tables

**Table 1:** Chemical and microbial compositions of perennial oat.

	Heading Stage	Flowering Stage	SEM	*p*-Value
DM, %FM	28.43	34.17	2.35	<0.001
WSC, %DM	4.89	6.12	0.89	<0.001
CP, %DM	8.11	8.96	0.42	<0.001
NDF, %DM	54.69	47.96	3.17	<0.001
ADF, %DM	32.11	27.25	2.48	<0.001
LAB, log_10_ cfu/FM	5.71	4.26	0.26	<0.001
Aerobic bacteria, log_10_ cfu/FM	5.98	6.44	0.49	<0.001
Coliform bacteria, log_10_ cfu/FM	5.41	6.18	0.33	<0.001
Yeasts, log_10_ cfu/FM	6.17	5.31	0.28	<0.001

ADF, acid detergent fiber; CP, crude protein; DM, dry matter; FM, fresh matter; LAB, lactic acid bacteria; SEM, standard error of mean; WSC, water soluble carbohydrates.

**Table 2:** Chemical composition of perennial oat silages treated without (CK) or with local LAB inoculant (IN1), commercial LAB inoculant (IN2), and sodium benzoate (BL).

	DM	DMR	WSC	CP	NDF	ADF
	% FM)	%DM				
Heading stage (H)						
CK	30.66 de	93.24 c	1.12	7.26	62.03	36.57
IN1	31.83 d	95.64 b	1.21	7.52	63.49	36.89
BL	30.92 de	97.35 a	1.16	7.61	57.60	33.65
IN2	30.18 e	92.20 c	1.18	7.31	62.31	36.33
Flowering stage (F)						
CK	38.82 ab	95.35 b	1.20	7.75	50.55	29.97
IN1	37.68 b	97.67 a	1.16	8.02	50.76	30.19
BL	35.64 c	96.94 a	1.18	8.13	46.64	27.45
IN2	39.20 a	93.11 c	1.25	7.82	50.61	29.79
SEM	0.57	0.32	0.06	0.05	0.98	0.55
Maturity stage (M)						
H	30.90	94.61	1.18	7.43 b	61.36 a	35.86 a
F	37.83	95.77	1.20	7.93 a	49.64 b	29.35 b
Additives (A)						
CK	33.15	94.29	1.72	7.51 c	56.29 a	33.27 a
IN1	34.75	96.65	1.18	7.77 a	57.13 a	33.54 a
BL	34.87	97.15	1.17	7.87 a	52.12 b	30.55 b
IN2	34.69	92.66	1.22	7.57 b	56.46 a	33.06 b
Significant (*p*-value)						
M	<0.001	0.001	0.295	<0.001	<0.001	<0.001
A	0.005	<0.001	0.392	<0.001	<0.001	<0.001
M×A	<0.001	0.019	0.279	0.979	0.712	0.962

ADF, acid detergent fiber; CP, crude protein; DM, dry matter; DMR, dry matter recovery; SEM, standard error of mean; WSC, water soluble carbohydrates. Values with different letters in the same column are significantly different (*p* < 0.05).

**Table 3:** Aerobic stability and microbial compositions of perennial oat silages treated without (CK) or with local LAB inoculant (IN1), commercial LAB inoculant (IN2), and sodium benzoate (BL).

	Aerobic Stability	LAB	Aerobic Bacteria	Coliform Bacteria	Yeasts
	h	Log_10_ cfu/g FM			
Heading stage (H)					
CK	74.67 f	6.57 c	7.31 a	4.31 b	4.37 b
IN1	157.33 a	8.27 a	3.41 c	2.26 e	3.25 e
BL	144.00 b	5.63 d	3.08 c	1.48 f	2.40 f
IN2	93.33 e	8.59 a	4.57 b	3.50 d	3.28 e
Flowering stage (F)					
CK	54.00 g	5.56 d	7.00 a	4.84 a	5.32 a
IN1	108.00 d	7.53 c	4.82 b	4.07 c	4.60 c
BL	132.67 c	5.33 d	3.40 c	3.53 d	2.33 f
IN2	97.33 e	7.57 c	4.70 b	4.51 b	3.85 d
SEM	5.26	0.15	0.24	0.17	0.16
Maturity stage (M)					
H	117.33	6.77	4.59	2.89	3.33
F	98.00	6.25	4.98	4.24	4.03
Additives (A)					
CK	64.33	6.07	7.15	4.58	4.85
IN1	132.67	7.90	4.12	3.17	3.93
BL	138.33	5.48	3.24	2.51	2.37
IN2	95.33	6.58	4.63	4.01	3.57
Significant (*p*-value)					
M	<0.001	<0.001	0.001	<0.001	<0.001
A	<0.001	<0.001	<0.001	<0.001	<0.001
M×A	<0.001	0.016	<0.001	<0.001	<0.001

LAB, lactic acid bacteria; SEM, standard error of mean. Values with different letters in the same column are significantly different (*p* < 0.05).

**Table 4:** Fermentation parameters of perennial oat silages treated without (CK) or with local LAB inoculant (IN1), commercial LAB inoculant (IN2), and sodium benzoate (BL).

	Final pH	Lactate	Acetate	Propionate	Butyrate	Ammonia-N
		%DM				%TN
Heading stage (H)						
CK	4.50 e	0.83 b	0.20 ef	0.08 d	0.04 bc	13.73 a
IN1	4.23 f	1.28 a	1.51 a	0.36 a	—	9.19 c
BL	4.55 e	0.58 c	0.08 g	0.02 e	—	7.81 e
IN2	4.57 e	0.80 b	0.67 c	0.14 c	0.06 ab	10.73 b
Flowering stage (F)						
CK	5.31 a	0.41 de	0.13 fg	0.06 de	0.08 a	9.99 bc
IN1	4.71 d	0.85 b	1.23 b	0.31 b	0.03 bc	9.03 cd
BL	5.19 b	0.38 e	0.22 e	0.09 d	0.02 c	8.01 de
IN2	4.98 c	0.51 cd	0.38 d	0.08 d	0.08 a	11.15 b
SEM	0.06	0.05	0.08	0.02	0.00	0.29
Maturity stage (M)						
H	4.46	0.87	0.61	0.15	—	10.36
F	5.05	0.54	0.49	0.13	0.05	9.55
Additives (A)						
CK	4.90	0.62	0.17	0.07	0.06	11.86
IN1	4.47	1.07	1.37	0.34	—	9.11
BL	4.87	0.48	0.15	0.06	—	7.91
IN2	4.77	0.66	0.52	0.11	0.07	10.94
Significant (*p*-value)						
M	<0.001	<0.001	<0.001	0.062	0.005	0.007
A	<0.001	<0.001	<0.001	<0.001	0.001	<0.001
M×A	<0.001	0.034	<0.001	0.001	0.190	<0.001

DM, dry matter; TN, total nitrogen. Values with different letters in the same column are significantly different (*p* < 0.05).

**Table 5:** In vitro ruminal fermentation parameters and DM digestibility of perennial oat silages treated without (CK) or with local LAB inoculant (IN1), commercial LAB inoculant (IN2), and sodium benzoate (BL).

	A1	A2	A	C	GP	IVDMD
	mL/g	mL/g	mL/g	mL/h	mL/g DM	g/kg DM
Heading stage (H)						
CK	2.49 c	42.48 d	44.98 e	0.04	41.19	43.57
IN1	4.15 a	47.36 a	51.51 a	0.06	45.18	46.25
BL	2.67 c	46.38 ab	49.05 bc	0.04	45.80	46.13
IN2	3.51 b	44.80 c	48.31 c	0.05	41.13	43.33
Flowering stage (F)						
CK	2.31 c	45.99 b	48.30 c	0.04	43.50	45.79
IN1	3.32 b	46.11 b	49.43 b	0.05	47.52	47.63
BL	2.65 c	46.68 ab	49.33 b	0.03	46.88	46.76
IN2	2.47 c	44.35 c	46.82 d	0.05	43.06	44.25
SEM	0.10	0.24	0.29	0.00	0.38	0.25
Means of maturity stage (M)						
H	3.21	45.25	48.46	0.05	43.33 b	44.82 b
F	2.69	45.78	48.47	0.04	45.24 a	46.11 a
Means of additives (A)						
CK	2.40	44.24	46.64	0.04 bc	42.35 b	44.68 b
IN1	3.73	46.73	50.47	0.06 a	46.35 a	46.94 a
BL	2.66	46.53	49.19	0.03 c	46.34 a	46.45 a
IN2	2.99	44.57	47.57	0.05 ab	42.10 b	43.79 b
Significant(*p*-value)						
M	<0.001	0.039	0.973	0.332	<0.001	0.004
A	<0.001	<0.001	<0.001	0.004	<0.001	<0.001
M × A	0.004	<0.001	<0.001	0.337	0.595	0.496

A1 is the gas production from the immediately soluble fraction (ml); A2 is the gas production from the insoluble fraction (mL); C is the gas production rate constant (mL/h); A1 + A2 is the potential gas production (mL); DM, dry matter; GP, as production; IVDMD, in vitro dry matter digestibility. Values with different letters in the same column are significantly different (*p* < 0.05).

## Data Availability

Data are available on request from the corresponding author with reasonable reason.
